# X chromosome-wide association studies in neurological disorders: uncovering the hidden influence of the X chromosome

**DOI:** 10.3389/fgene.2025.1650259

**Published:** 2025-07-30

**Authors:** Kathryn Step, Thiago Peixoto Leal, Walaa A. Kamel, Emily Waldo, Soraya Bardien, Ignacio F. Mata

**Affiliations:** ^1^ Division of Molecular Biology and Human Genetics, Faculty of Medicine and Health Sciences, Stellenbosch University, Cape Town, South Africa; ^2^ South African Medical Research Council Centre for Tuberculosis Research, Stellenbosch University, Cape Town, South Africa; ^3^ Genomic Medicine, Lerner Research Institute, Cleveland Clinic Foundation, Cleveland, OH, United States; ^4^ Department of Neurology, Faculty of Medicine, Beni-Suef University, Beni-Suef, Egypt; ^5^ Division of Molecular Biology and Human Genetics, Faculty of Medicine and Health Sciences and South African Medical Research Council/Stellenbosch University Genomics of Brain Disorders Research Unit, Stellenbosch University, Cape Town, South Africa

**Keywords:** Parkinson’s disease, X-chromosome-wide association study, neurodegenerative disease, XWAS, association analysis, sex bias, susceptibility variants

## Abstract

X chromosome-wide association studies (XWAS) have identified susceptibility variants for various neurodegenerative and neurodevelopmental diseases. The unique characteristics of the chromosome require more complex analytical approaches than standard genome-wide association studies. Over the past 2 decades, refined XWAS methods have better accounted for this biology. Given that many neurological diseases show sex-biased prevalence, XWAS offers a valuable framework for investigating sex-specific genetic contributions. This review summarizes published neurological XWAS (*N* = 10), highlighting methodological approaches. Despite the challenges of genetic analyses for the X chromosome, XWAS remains a key approach for studying its role in disease mechanisms.

## Introduction

**FIGURE 1:**
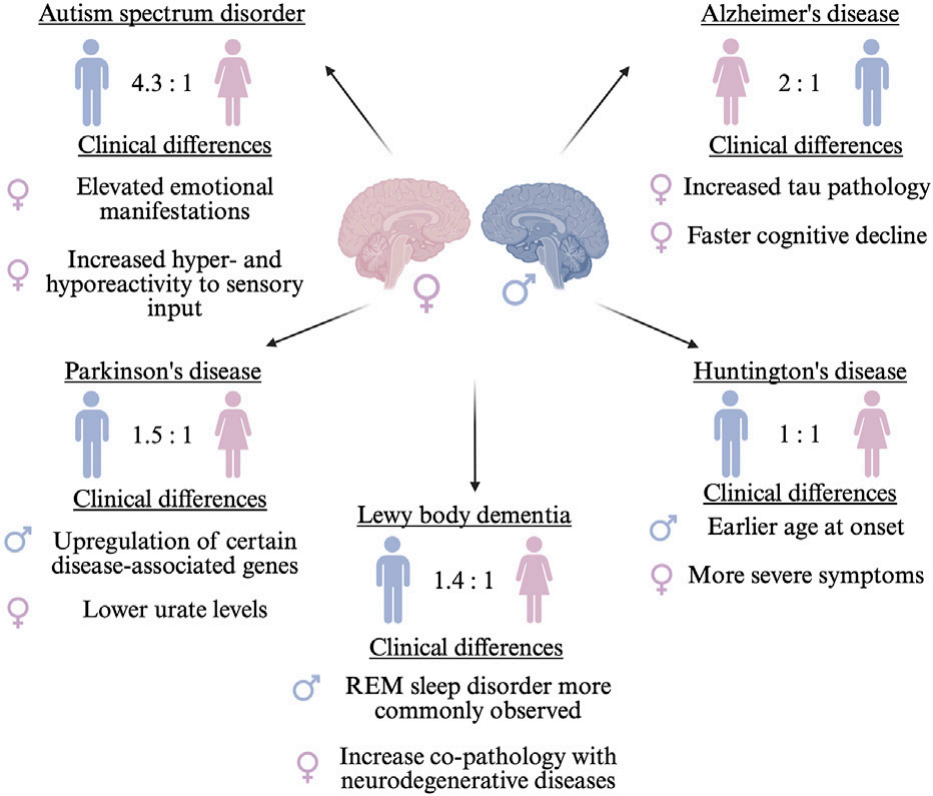
Sex differences observed in neurological disorders including prevalence ratios and clinical manifestations. The sex indicated on the left has the higher prevalence ratio. ♂ indicates male and ♀ indicates female.

Sex differences impact brain morphology and neural connections, starting during embryonic development and continuing throughout growth (Savica et al., 2013; Ullah et al., 2019). Sex hormones play a major role in the structural organization and brain function contributing to disparities in neurological disorders, including clinical phenotype, diagnostic timelines, and therapeutic approaches (Figure 1) (Göttgens et al., 2020; Arabia et al., 2022). In addition to hormonal influences, which may have a protective effect (Siddiqui et al., 2016), genetic mechanisms play a pivotal role in sex-biased neurological disease vulnerability due to the unique biology of the X chromosome (Xchr), which harbors numerous brain-related genes (Jiang et al., 2025). Importantly, sex-based differences in disease risk and phenotype may vary by ancestry (GBD, 2021 Nervous System Disorders Collaborators, 2024), highlighting the need for diverse studies.

Alzheimer’s disease (AD) is twice as prevalent in females (Ferretti et al., 2020; Beam et al., 2018), which may be somewhat associated with differences in lifespan; however, females may have more neurofibrillary tangle pathology and elevated cerebrospinal fluid total tau and Aβ42 levels compared to males (Koran et al., 2017). Additionally, females show a faster cognitive decline (Ferretti et al., 2018). Conversely, Parkinson’s disease (PD), is about 1.5 times more common in males (Ben-Shlomo et al., 2024), which may be driven by underlying biology, environmental exposures, and behavioral factors (Savica et al., 2013). For instance, certain PD-related genes (*SNCA* and *PINK1*) show higher expression in males (Crispino et al., 2020), while lower urate levels and reduced coffee consumption increases female risk (Pottmeier et al., 2024). Moreover, Lewy body dementia (LBD) also displays sex differences. Although females are underrepresented in LBD cohorts, likely reflecting higher diagnostic rates in males rather than true prevalence differences (Irwin et al., 2017), certain clinical features and comorbidities show sex-specific patterns. For instance, REM sleep behaviour disorder is more frequently observed in males with LBD (Postuma et al., 2019), while LBD co-pathology with AD or PD appears more common in females (Ferman et al., 2020).

Sex differences are especially evident in symptom profiles and disease progression, with notable variations observed across different neurodegenerative disorders. These differences are particularly well-characterized in PD (Hanff et al., 2025), where females are more likely to experience anxiety, depression, fatigue, dysphagia, constipation, and pain (Bovenzi et al., 2024). Additionally, females exhibit greater postural instability and more rapid disease progression (Picillo et al., 2022). Similar sex-based differences in symptoms and progression have been noted in AD and other neurodegenerative conditions. Furthermore, clinical manifestations and cognitive assessment profiles vary between the sexes (Aggarwal and Mielke, 2023). Treatment and biomarker profiles (e.g., blood and neuroimaging) have been demonstrated to vary between the sexes. Numerous studies indicate a greater prevalence of parkinsonism in males compared to females with LBD (Bayram et al., 2021).

While it is recognized that CAG repeat instability occurs in male intergenerational transmission of Huntington’s disease (HD), the sex-related changes in the natural history of HD remain poorly understood, potentially due to its autosomal dominant inheritance, often considered sex-independent (Zie and lonka, 2018). Nonetheless, sex-specific modifiers of age of onset of HD have been reported: males with the *APO*E ε2ε3 genotype have an earlier onset compared to females (Meoni et al., 2020), and *PPARGC1A* variants show an earlier motor onset in males (Weydt et al., 2014). Conversely, females with HD exhibit more severe motor, cognitive, and depressive symptoms (Risby-Jones et al., 2024), although the overall rate of symptom progression is similar between the sexes over time (Hentosh et al., 2021).

Conversely, Autism Spectrum Disorder (ASD), a neurodevelopmental disorder marked by impairments in social communication and the manifestation of restricted interests and repetitive behaviors, is one of the most common childhood disorders among males, with a male-to-female ratio of 4.3:1 (Werling, 2016), although this disparity may partly reflect a diagnosis bias rather than true prevalence, as females often exhibit better masking of symptoms and remain underdiagnosed (McCrossin, 2022). It has been hypothesized that females may be relatively protected from developing ASD, potentially requiring a greater cumulative burden of genetic and/or environmental risk factors before symptoms manifest (Wigdor et al., 2022). However, a distinction in core symptoms is observed between the sexes. Adult females with ASD scored significantly higher than males on measures of hyper- and hyporeactivity to sensory input (Gesi et al., 2021). Moreover, females with ASD exhibited a greater propensity for internalizing issues, including sadness, suicide risk, anxiety, and other emotional disturbances (So et al., 2021).

## Biological characteristics of the X chromosome

The Xchr accounts for approximately 5% of the human genome (Sun et al., 2023) and comprises 156 million base pairs (Gorlov and Amos, 2023). Several biological features distinguish the Xchr from autosomes. Females have two Xchrs, while males have one X and one Y chromosome. To balance gene expression between the sexes, dosage compensation occurs through X-inactivation, which silences many genes on one Xchr in females (Gartler and Goldman, 2001). This process excludes the pseudoautosomal regions, which are regions of the X and Y chromosomes that recombine during male meiosis (Lukin et al., 2024). At a population level, X-inactivation can be skewed, random, or absent, adding complexity to the regulation and interpretation of X-linked gene expression (Sun et al., 2023). Recombination occurs along the full length of both Xchrs in females (Le Guen et al., 2021), but only within the pseudoautosomal regions in males (Sun et al., 2023). These differences contribute to increased evolutionary influences, such as bottlenecks, natural selection, and sex-bias admixture (Mendes et al., 2025), on the Xchr in comparison to autosomes, with females under greater selective pressure for Xchr mutations (Gorlov and Amos, 2023; Le Guen et al., 2021; Leal et al., 2023).

## X chromosome genetic variants

Although the Xchr has been studied in the context of neurological diseases, it has received considerably less attention than the autosomes in genetic research. Family-based linkage analyses and candidate gene studies have identified candidate loci on the Xchr for neurological disorders such as PD (Pankratz et al., 2002; Klein and Westenberger, 2012) and ASD (Holt et al., 2010; Scala et al., 2025). However, these linkage studies have been conducted predominantly in European cohorts, limiting the understanding of X-linked genetic risk in other ancestries, where genetic associations may differ. For example, the prevalence of AD and related dementias, including LBD, is higher in African American and Latin American populations, with differences in genetic risk factors such as *APOE ε4* (Bayram et al., 2024; Matthews et al., 2019).

Over the past 2 decades, genome-wide association studies (GWAS) have been used to identify risk loci contributing to phenotypic traits (Abdellaoui et al., 2023), including susceptibility to neurological diseases (Tan et al., 2014; He et al., 2024). However, the name of this approach can be misleading, as GWAS often focus exclusively on the autosomes, excluding the sex chromosomes altogether (Sun et al., 2023). Consequently, Xchr hits in GWAS remain approximately six times fewer than autosomal hits, based on comparisons of GWAS-identified single-nucleotide polymorphism densities (hits per megabase) across chromosomes (Gorlov and Amos, 2023), because the Xchr is frequently excluded from analyses. This discrepancy is largely due to the aforementioned chromosome’s unique characteristics, such as hemizygosity in males, X-inactivation in females, and lower variant density, which complicate analysis and often lead to its exclusion from standard GWAS pipelines (Leal et al., 2023). As a result, many potential X-linked associations have likely been missed. This underscores the importance of approaches specifically designed to account for the Xchr’s unique biology. As analytical methods continue to evolve, routinely integrating the Xchr into GWAS holds promise for uncovering previously overlooked genetic contributions to complex traits.

## Analytical overview of X chromosome-wide association studies

The autosomal quality control (QC) typically includes: (i) removing missing data for variants and individuals, (ii) checking for sex discrepancies, (iii) removing ambiguous variants, (iv) removing multiallelic, duplicated, monomorphic and probe polymorphism variants, (v) removing related individuals, (vi) filtering based on minor allele frequency, and (vii) removing variants deviating from Hardy-Weinberg equilibrium (HWE) (Le Guen et al., 2021; Mendes et al., 2025; Leal et al., 2023). Typically, variants are then separated into the non-pseudoautosomal and pseudoautosomal regions using a reference file, and the analysis excludes the latter, as they behave more like autosomes and often present genotyping challenges. The next step is to conduct Xchr specific QC, including: (i) selection or exclusion of individuals based on ancestry or relatedness, (ii) removing variants with differential missingness between sexes, (iii) assigning “missing data” to heterozygous SNPs in males, and (iv) removing variants that failed HWE in female controls (Leal et al., 2023; Bayram et al., 2024; Belloy et al., 2024). Importantly, including males in HWE for Xchr QC may lead to their exclusion owing to their hemizygosity (Gorlov and Amos, 2023). The remaining variants can be checked against a reference panel for minor allele frequencies consistency, and to confirm that only non-palindromic variants are retained (Le Guen et al., 2021). Once QC is complete, the data should be imputed.

Association studies commonly include principal components (PCs) as covariates to control for population stratification within study cohorts (Peloso and Lunetta, 2011), and can be used to define a more homogeneous cohort for analysis (Le Guen et al., 2021). In the context of XWAS, PCs can be calculated using autosomal variants, Xchr variants, or a combination of both, depending on the study design and objective (Leal et al., 2023). However, in multi-way admixed populations, special care must be taken when analyzing population structure. A more stringent and nuanced approach is needed to accurately account for substructure, as inadequate correction may lead to spurious associations and false-positive results (Peloso and Lunetta, 2011). To address this, projected PCs (calculated by projecting study samples onto a reference panel) can be used to more effectively capture and control for the axes of variation contributing to population structure.

A XWAS can be conducted in a sex-stratified manner to identify disease-associated loci specific to either females or males. Typically, standard logistic regression models are applied with the appropriate covariates to account for variability within the dataset (Le Guen et al., 2021), using association testing software such as PLINK (Chang et al., 2015; Purcell et al., 2007). The common covariates include age, sex, PCs, and additional non-biological sources of variation (Belloy et al., 2024). To account for dosage differences between the sexes on the Xchr, PLINK provides the*--xchr-model* flag, which enables various modes to model sex-specific genotypic variation and dosage compensation (Purcell et al., 2007). However, this is applicable only when analyses are not performed in a sex-stratified manner. To obtain the overall XWAS results and identify sex-independent risk loci, a meta-analysis of the stratified sex-results can be performed (Mendes et al., 2025; Leal et al., 2023). This is commonly implemented using meta-analysis softwares such as GWAMA (Mägi and Morris, 2010), which is preferred due to its ability to report heterogeneity and differentiation between the sexes (Le Guen et al., 2021; Simmonds et al., 2024).

Association studies, typically GWAS, use a standard threshold to indicate genome-wide significance, calculated by dividing 0.05 by the number of independent tests across the genome (5 × 10^−8^) (Fadista et al., 2016). However, in XWAS, only one chromosome is included, meaning a new significance threshold must be calculated, typically using Bonferroni correction (0.05/number of effective tests) (Mendes et al., 2025; Leal et al., 2023; Bayram et al., 2024). As mentioned above, there are a number of approaches one can use when performing XWAS, including sex-stratified analysis and meta-analysis. Each of these approaches yields a different number of effective tests, ultimately resulting in a different significance threshold calculation for each scenario (Mendes et al., 2025).

## Main findings from published studies

To date, there are 23 published XWAS (Supplementary Table S1), with ten focused on neurological disorders (Table 1; Supplementary Table S2), nine of which were published in the last 5 years. These include AD (n = 4), ASD (n = 2), PD (n = 2), HD (n = 1), and LBD (n = 1). However, only one XWAS on PD (Leal et al., 2023) and one on AD (Wang et al., 2023), included individuals of non-European ancestry. Over time, XWAS methodologies have evolved to account for the unique biology of the Xchr, with recent studies incorporating both sex-stratified analyses and meta-analytic approaches to increase power and better capture sex-specific effects.

**TABLE 1:** Overview of the published X chromosome-wide association studies on neurological disorders.

Disorder	Participant information	Method	Main finding	References
2011				
ASD	7,505 cases, 6,472 controls. European (100%)	XWAS using modified X-APL and PLINK; sex-stratified and combined meta-analysis with SimpleM-adjusted threshold (p < 6.25 × 10^−6^)	Rs17321050 in *TBL1X* gene reached chromosome-wide significance in meta-analysis and in male joint analyses	Chung et al. (2011)
2021				
HD	8,963 cases, 0 controls. European (100%)	XWAS with GEMMA (LMM) on residual age-at-onset; meta-, sex-stratified, dichotomous, and conditional analyses	No genome-wide hits; suggestive loci near *MSN*, *VSIG4*, *PTCHD1-AS*, *GRIA3*, and regions in Xq21.22/Xp22.31	Hong et al. (2021)
PD	11,142 cases, 280,164 controls, 5,379 proxy cases. European (100%)	Sex-stratified XWAS using PLINK and GWAMA; colocalization with brain eQTLs and imaging via BOLT-LMM.	Rs7066890 and rs28602900 genome-wide significant; rs28602900 replicated and is an eQTL for *RPL10*	Le Guen et al. (2021)
2023				
AD	3,079 cases, 0 controls. European (89.52%), African American (4.46%), Hispanic (3.30%), Other (2.72%)	Non-stratified cross-sectional and longitudinal XWAS using mixed models in R; Bonferroni-corrected threshold (p < 2.54 × 10^−6^)	15 significant SNPs associated with biomarkers near genes including *DMD*, *TBX22*, *TENM1*, and *AFF2*	Wang et al. (2023)
PD	925 cases, 1505 controls. Latinos (100%)	Meta- and sex-stratified XWAS using Firth logistic regression in PLINK; analysis-specific significance thresholds applied	Eight significant loci; *rs525496* replicated; confirmed prior hit *rs28602900*	Leal et al. (2023)
2024				
AD	115,841 cases, 613,671 controls. European (100%)	Three methods to model X-inactivation in females; adjusted for population structure; sex-stratified analysis; threshold p < 1.6 × 10^−6^	Seven significant loci identified including *FRMPD4*, *NLGN4X*, *GRIA3*, *DMD*, *WNK3*, *PJA1*, and *DACH2*	Le Borgne et al. (2024)
AD	56,172 cases, 82,386 proxy cases, 1,013,726 controls. European (100%)	Comprehensive XWAS modeling random X-inactivation; adjusted for covariates; meta-analysis and colocalization to prioritize genes; non-stratified analysis	Six significant loci including *SLC9A7* (top hit rs2142791), *NLGN4X*, *MIDI*, *ZNF280C*, *ARGRG4*, and *MTM1*	Belloy et al. (2024)
AD	1,970 cases, 1,113 controls. European (100%)	Sex-stratified and combined XWAS meta-analyses with FDR correction; gene-based MAGMA and expression analyses to assess biological relevance	Four replicated loci (*DDX53*, *IL1RAPL1*, *TBX22*, *SH3BGRL*); rs5913102 significant in meta-analysis, rs5944989 in females	Simmonds et al. (2024)
LBD	2,591 cases, 4,391 controls. European (100%)	Haplotype and single-variant XWAS with PLINK; adjusted by PCs via AIC model; APOE ε4 conditioning, MAGMA, rare variant tests, and eQTL colocalization	One female-specific risk locus (rs141773145) in *MAP3K15*; no significant male or meta-analysis findings	Bayram et al. (2024)
2025				
ASD	6,873 cases, 8,981 controls. European (100%)	XWAS using GENESIS PCA, PLINK logistic regression, GWAMA meta-analysis, and gene-sex interaction (sdMAF); variant and CNV calling; brain expression from BrainSpan; chromosome-wide thresholds applied	59 significant variants: 27 male-specific, 5 female-specific, 1 combined, 9 meta-analysis; 17 shared across tests	Mendes et al. (2025)

A more comprehensive summary is provided in Supplementary Table S2. Studies are current as of 06/05/2025. Genome-wide significance set at p < 5 × 10^−8^ and suggestive significance at p < 1 × 10^−5^. Chromosome-wide significance thresholds are unique to each study and specified accordingly. AD, Alzheimer’s disease; ASD, autism spectrum disorder; AIC, akaike information criterion; CNV, copy number variant; eQTL, expression quantitative trait loci; FDR, false discovery rate; GWAMA, Genome-Wide Association Meta-Analysis; HD, Huntington’s disease; LBD, lewy body dementia; LMM, linear mixed model; MAGMA, Multi-marker Analysis of GenoMic Annotation; PCA, principal component analysis; PD, Parkinson’s disease; QTL, quantitative trait loci; SNP, single nucleotide polymorphism; X-APL, X-chromosome Analysis of Pedigree and Linkage; XWAS, X chromosome-wide association study.

The first XWAS for a neurological disorder was published in 2011, linking risk loci on the Xchr with ASD (Chung et al., 2011). This study (*N* = 13,977) used both sex-stratified analysis and meta-analysis to successfully identify a risk locus (rs17321050) in the *TBL1X* gene, reaching chromosome-wide significance. The next ASD XWAS was done by Mendes et al. (2025), using whole-genome sequencing data and a larger cohort (*N* = 15,854). This more recent study analyzed over 400,000 X-linked variants to identify 59 significant associations (Mendes et al., 2025). The advancement of XWAS approaches over the past decade is reflected here, demonstrating improved statistical power and a more comprehensive exploration of the Xchr.

A decade after the first XWAS was published (Chung et al., 2011), the next neurological disorder to undergo this analysis was HD (Hong et al., 2021). This XWAS aimed to identify X-linked variants modifying age-at-onset in individuals with HD (*N* = 8,963), separating cases based on CAG repeat lengths (Hong et al., 2021). No genome-wide significance hits were observed for the sex-stratified and meta-analysis approaches. However, the meta-analysis identified four regions of suggestive significance, while the male- and female-stratified analyses each revealed three suggestive loci.

The first PD XWAS (*N* = 296,685) was published in 2021 by Le Guen et al., identifying two loci that reached genome-wide significance (rs7066890 and rs28602900), the latter of which was replicated in an independent dataset and shown to colocalize with an expression quantitative trait locus (eQTL) for *PRL10* expression in the putamen and other brain regions (Le Guen et al., 2021). A Latin American PD cohort (*N* = 2,430) was the focus for the second PD XWAS (Leal et al., 2023). This study adapted the Le Guen et al. (2021) pipeline to better suit an admixed study cohort by modifying the autosomal and Xchr QC as well as adding population structure analysis. Eight regions were associated with PD, where one novel locus (rs525496) was successfully replicated across independent cohorts. Moreover, the study confirmed the previously reported rs28602900 association by Le Guen and colleagues (Le Guen et al., 2021).

To date, most published XWAS have focused on AD, with one AD XWAS published in 2023 (Wang et al., 2023) and three in 2024 (Belloy et al., 2024; Simmonds et al., 2024; Le Borgne et al., 2024). The first AD XWAS (*N* = 3,079) aimed to assess associations between X-linked variants and 16 AD quantitative biomarkers (Wang et al., 2023). In total, 15 statistically significant loci were identified in or near eight genes: *DMD*, *TBX22*, *LOC101928437*, *TENM1*, *SPANXN1*, *ZFP92*, *RAC1P4*, and *AFF2*. The next AD XWAS (*N* = 729,512) employed three independent AD studies and included both a sex-stratified analysis and meta-analysis (Simmonds et al., 2024). Four suggestively associated genes were identified across at least two studies: *DDX53* (rs12006935), *IL1RAPL1* (rs6628450, rs137983810), *TBX22* (rs5913102), and *SH3BGRL* (rs186553004, rs113157993), with rs5913102 achieving chromosome-wide significance in the meta-analysis. For the sex-stratified analysis, one locus (rs5944989) reached chromosome-wide significance in females. Moreover, in a non-stratified analysis, Belloy et al. (2024) performed a meta-analysis of multiple AD studies in individuals of European ancestry (*N* = 1,152,284). Here, six loci reached chromosome-wide significance, with four passing suggestive genome-wide significance (Belloy et al., 2024). The latest AD XWAS to date (*N* = 3,083), investigated three approaches to account for Xchr inactivation states, specifically random inactivation, skewed inactivation, and escape from inactivation (Le Borgne et al., 2024). Seven loci were significant at a chromosomal level, highlighting potential X-linked regions of interest.

The final neurological disorder with an XWAS analysis is LBD (*N* = 6,982), where the first and only XWAS was conducted by Bayram et al. (2024). The study included a meta-analysis and sex-stratified approach, with a chromosome-wide significance threshold. One significant risk locus (rs141773145) was identified in the first intron of *MAP3K15* among female cases, conditioned for *APOE ε4* dosage (Bayram et al., 2024).

Collectively, XWAS of neurological disorders have highlighted several X-linked loci of interest, though replication remains limited and representation of non-European populations is sparse. Methodological advancements, particularly in sex-stratified and meta-analytic approaches, have improved discovery power. Future studies should prioritize diverse cohorts and functional validation of identified variants.

## Limitations, current gaps in research, and future directions

XWAS can be performed using genotyping or sequencing data; however, when relying on genotyping arrays, genotyping and imputation quality for the Xchr are generally lower than for autosomes (Leal et al., 2023). This can lead to reduced variant coverage, lower call rates, and decreased imputation quality and accuracy (Le Borgne et al., 2024), all of which impact the reliability of the data used in XWAS. Moreover, some studies have reported no significant findings in female-stratified analyses, possibly due to skewed data resulting from X-inactivation and a smaller female sample size in comparison to males (Chung et al., 2011). These disparities may stem from recruitment bias or inherent sex differences in disease prevalence. In some cases, males were intentionally prioritized for inclusion to reduce the “noise” introduced by the Xchr biology (Le Guen et al., 2021). However, it is essential to include sex-balanced cohorts in this analysis to aid in the identification of sex-specific risk variants. Furthermore, many neurological disorder datasets used in the published XWAS are derived from family-based studies, which often lack sex-matched case-control recruitment, ultimately affecting the reliability of association results (Belloy et al., 2024; Chung et al., 2011).

Study cohorts vary in size, with previously underrepresented populations in genetics research often comprising significantly smaller sample sizes (Leal et al., 2023). This limitation reduces the statistical power of XWAS. Furthermore, many studies, even those involving European populations, have reported difficulty identifying independent cohorts for replication (Bayram et al., 2024). This challenge is even more pronounced for ancestrally diverse populations, where replication datasets are often scarce or nonexistent. In the context of XWAS, replication is further complicated by the need for sex-stratified analyses, which can divide already limited datasets, making it difficult to set aside separate discovery and validation cohorts (Bayram et al., 2024). The lack of replication is also partly due to the novelty of this analytical approach, as only a limited number of XWAS have been published for specific neurological diseases (Bayram et al., 2024; Hong et al., 2021). Additionally, the inclusion of diverse ancestries requires more complex and customized pipelines for quality control and data harmonization (Leal et al., 2023). The limited ancestral diversity in published studies continues to restrict opportunities for robust replication and the generalizability of findings.

Finally, the availability and accuracy of *in silico* prediction tools for post-XWAS analyses can significantly influence the interpretation of the biological relevance of identified variants. One challenge is that index variants may lie several hundred kilobases away from the nearest protein-coding gene, complicating functional annotation (Le Borgne et al., 2024). Some evidence suggests that the Xchr may have a lower overall density of functional variants (Gorlov and Amos, 2023). While this may partially reflect the historical exclusion of the Xchr from many genetic studies, it underscores the need for continued research and improved annotation tools, particularly if XWAS findings are to inform clinical applications and risk prediction models.

## Best practice approaches for X chromosome-wide association studies

The QC and analysis of the Xchr in association studies require approaches that differ slightly from those used for autosomes due to its unique characteristics. Given the differences in ploidy and X-inactivation, sex-stratified analyses followed by a meta-analysis are generally recommended to address these complexities and facilitate the detection of sex-specific risk loci. Additionally, an adjusted p-value significance threshold should be considered based on the number of independent tests across the Xchr. Finally, to support open science and replication efforts, summary statistics from XWAS should be made publicly available whenever possible.

## Concluding remarks

Advancements in analytical approaches have facilitated the inclusion of the Xchr in association studies. Given the apparent sex differences in several diseases it is imperative that this chromosome should not routinely be excluded. Encouragingly, as more XWAS are conducted, an increasing number of risk loci are being replicated, reinforcing the reliability of these approaches. Continued progress in bioinformatics and routine inclusion of this chromosome in analyses are vital to identify risk loci that previous association studies may have excluded. Ultimately, prioritizing the Xchr in future association studies will be critical for uncovering the full spectrum of genetic risk factors for complex diseases.
