# Supplementary material for: X chromosome-wide association studies in neurological disorders: uncovering the hidden influence of the X chromosome
Source: Front Genet. 2025 Jul 30;16:1650259. doi: 10.3389/fgene.2025.1650259 (PMC12343220; doi:10.3389/fgene.2025.1650259)
Supplement: Supplementary file 1 [file DataSheet1.pdf]

X chromosome-wide association studies in neurological disorders: Uncovering the hidden influence of the X chromosome

Kathryn Step, Thiago Peixoto Leal, Walaa A. Kamel, Emily Waldo, Soraya Bardien, Ignacio F. Mata

Table of contents

Supplementary Tables.......... 2

Supplementary Table 1: List of published X chromosome-wide association studies to date.......... 2

Supplementary Table 2: Overview of the published X chromosome-wide association studies on neurological disorders.......... 4

References.......... 8

Supplementary Tables

**Supplementary Table 1: List of published X chromosome-wide association studies to date.**			
**Year**	**Disease**	**Manuscript Title**	**Reference**
2011	Autism	An X chromosome-wide association study in autism families identifies TBL1X as a novel autism spectrum disorder candidate gene in males	(1)
2013	Graves' disease	An X chromosome-wide association analysis identifies variants in GPR174 as a risk factor for Graves' disease	(2)
2013	Follicular lymphoma	X chromosome-wide association study of follicular lymphoma	(3)
2014	Autoimmune disease & new XWAS software	Accounting for eXentricities: Analysis of the X Chromosome in GWAS reveals X-linked genes implicated in autoimmune diseases	(4)
2017	Asthma	Suggestive association between variants in IL1RAPL and asthma symptoms in Latin American children	(5)
2017	Inflammatory bowel disease	X chromosome-wide association study identifies a susceptibility locus for inflammatory bowel disease in Koreans	(6)
2019	Nasopharyngeal carcinoma	X-chromosome association study reveals genetic susceptibility loci of nasopharyngeal carcinoma	(7)
2021	Huntington's disease	Association Analysis of Chromosome X to Identify Genetic Modifiers of Huntington's Disease	(8)
2021	Parkinson's disease	Common X-Chromosome Variants Are Associated with Parkinson Disease Risk	(9)
2023	Celiac disease	Sex bias in celiac disease: XWAS and monocyte eQTLs in women identify TMEM187 as a functional candidate gene	(10)
2023	Assisted reproductive technologies	The X-factor in ART: does the use of assisted reproductive technologies influence DNA methylation on the X chromosome?	(11)
2023	Pulmonary disease	X chromosome associations with chronic obstructive pulmonary disease and related phenotypes: an X chromosome-wide association study	(12)

2023	Alzheimer's disease	X chromosome-wide association study of quantitative biomarkers from the Alzheimer’s Disease Neuroimaging Initiative study	(13)
2023	Parkinson's disease	X-Chromosome Association Study in Latin American Cohorts Identifies New Loci in Parkinson's Disease	(14)
2024	Lung cancer	A Statistical Testing Strategy Accounting for Random and Nonrandom (Skewed) X-Chromosome Inactivation Identifies Lung Cancer Susceptibility Loci among Smokers	(15)
2024	Lupus	Association of genetic variation on X chromosome with systemic lupus erythematosus in both Thai and Chinese populations	(16)
2024	Alzheimer's disease	Chromosome X-wide association study in case control studies of pathologically confirmed Alzheimer’s disease in a European population	(17)
2024	Lewy Body Dementia	Genetic analysis of the X chromosome in people with Lewy body dementia nominates new risk loci	(18)
2024	Macular degeneration	Identifying X-chromosome variants associated with age-related macular degeneration	(19)
2024	Alzheimer's disease	Role of the X Chromosome in Alzheimer Disease Genetics	(20)
2024	Alzheimer's disease	X‐chromosome-wide association study for Alzheimer’s disease	(21)
2025	Autism	Chromosome X-wide common variant association study in autism spectrum disorder	(22)
2025	Hypospadias	X-chromosome association study reveals genetic susceptibility loci of hypospadias in southern Chinese population	(23)

Legend: The list of all published XWAS, as of 12 May 2025. The search was conducted using PubMed and Scopus with the terms “XWAS,” “X-chromosome-wide association study,” and “X-chromosome-wide association analysis". The list excludes review articles and papers introducing XWAS software.

Supplementary Table 2: Overview of the published X chromosome-wide association studies on neurological disorders					
**Disease**	**Participant Information**	**Software used**	**Method**	**Main finding**	**Ref**
**2011**					
Autism Spectrum Disorder	7505 cases, 6472 controls. European (100%)	X-APL (modified), PLINK v1.9	An XWAS was conducted in autism families, including both sex-stratified and combined analyses. Meta-analysis was performed across cohorts, and a chromosome-wide significance threshold (p-value $< 6.25 \times 10^{-6}$) was calculated using the SimpleM method to account for multiple testing.	Rs17321050 in *TBL1X* gene reached chromosome-wide significance in meta-analysis and in male joint analyses.	(1)
**2021**					
Huntington's disease	8963 cases, 0 controls. European (100%)	GEMMA v0.98.1	A linear mixed model approach was applied to a phenotype represented by residual age-at-onset. Analyses included meta-analysis and sex-stratified XWAS using standard genome-wide significance thresholds. Additional analyses included dichotomous XWAS comparing extreme residual age-at-onset groups and conditional testing of the moesin region for association signals.	No genome-wide significant hits. For the meta-analysis, four suggestive significant regions at Xq12 near *MSN* and *VSIG4*, as well as rs10284174 at *PTCHD1-AS*. The male-stratified analysis showed three loci in *GRIA3*, while the female analysis showed three SNPs in Xq21.22 and one in Xp22.31.	(8)

Parkinson's disease	11142 cases, 280164 controls, 5379 proxy cases. European (100%)	PLINK v2.0, GWAMA, BOLT-LMM	The study employed a sex-stratified XWAS approach using logistic regression followed by fixed-effect meta-analysis to identify both sex-specific and sex-independent risk loci. Additional analyses included colocalization with brain eQTLs (GTEx, Braineac) and association with putamen volume.	Rs7066890 and rs28602900 reached genome-wide significance. The latter was replicated in an independent dataset and is an eQTL of *RPL10*. No significance observed in sex-stratified analysis.	(9)
**2023**					
Alzheimer's disease	3079 cases, 0 controls. European (89.52%), African American (4.46%), Hispanic (3.30%), Other (2,72%)	R v4.1.2, QMVtest, lme4, lmerTest	The study performed non-stratified cross-sectional and longitudinal XWAS on quantitative biomarkers, adjusting for socio-demographic factors, APOE4 dosage, and population structure using principal components. Analyses were conducted using mixed models and applying a Bonferroni-corrected chromosome-wide significance threshold (p-value $< 2.54\times10^{-6}$).	15 statistically significant SNPs were found to be associated with disease quantitative biomarkers located near or in the following genes: *DMD*, *TBX22*, *LOC101928437*, *TENM1*, *SPANXN1*, *ZFP92*, *RAC1P4*, and *AFF2*.	(13)
Parkinson's disease	925 cases, 1505 controls. Latinos (100%)	PLINK v2.0	Analyses included meta-analysis and sex-stratified XWAS performed using Firth's logistic regression, adjusting for age, sex, and population structure. We calculated chromosome-wide significance thresholds specific to each analysis (meta-analysis p-value $< 8.43\times10^{-5}$; male p-value $< 6.65\times10^{-5}$; female p-value $< 3.99\times10^{-5}$) to identify associated variants while controlling for demographic heterogeneity.	Identified eight chromosome-wide significant loci. One loci (rs525496) replicated in an independent cohort. Replicated previous XWAS hit (rs28602900).	(14)
**2024**					

Alzheimer's disease	115841 cases or proxy cases, 613671 controls. European (100%)	METAL, R v5.2.3, snpStats v3.4, SNPTEST v2.5.6, SAIGE v.1.0.9	Used three approaches to account of X chromosome inactivation in females, adjusting for population structure and study design, alongside conducting a sex-stratified analysis. They established a chromosome-wide significance threshold at p-value $< 1.6\times10^{-6}$ to determine significant associations.	Seven chromosome-wide significant loci were identified in *FRMPD4* (rs5933929), *NLGN4X* (rs4364769), *GRIA3* (rs191195705), *DMD* (rs5972406), *WNK3* (189139822), *PJA1* (rs771148434), and *DACH2* (1326297223).	(21)
Alzheimer's disease	56172 cases, 82386 proxy cases, 1013726 controls. European (100%)	GWAMA, R, GTEx	This study performed comprehensive XWAS modeling random X chromosome inactivation in females and adjusting for population structure, sex, age, and relatedness using mixed models across multiple cohorts. They combined results via meta-analysis, evaluated sex-specific effects and X-inactivation escape by comparing male and female effect sizes, and prioritized potential causal genes through colocalization with molecular QTL data. The analysis was not stratified. Used standard genome-wide significance thresholds.	Six loci reached chromosome-wide significance in *SLC9A7*, *NLGN4X*, *MIDI*, *ZNF280C*, *ARGRG4*, *MTM1*. One locus passed genome-wide significance with the top hit (rs2142791) in *SLC9A7*.	(20)
Alzheimer's disease	1970 cases, 1113 controls. European (100%)	PLINK v1.9, METAL, GWAMA, MAGMA v1.08	The study conducted sex-stratified and combined meta-analyses of XWAS, adjusting for age and principal components, with significance determined by false discovery rate correction (suggestive meta-analysis p-value $< 1.1 \times 10^{-3}$). Additionally, gene-based analyses were performed, and expression and protein interaction data from public and in-house RNA-seq datasets were leveraged to explore the biological relevance of identified risk genes.	Four loci in *DDX53*, *IL1RAPL1*, *TBX22*, and *SH3BGRL* were identified which replicated across at least the two analyses being the meta-analysis or sex-stratified analyses. rs5913102 achieved chromosome-wide significance in the meta-analysis and rs5944989 in the female analysis.	(17)

Lewy body dementia	2591 cases, 4391 controls. European (100%)	PLINK 1.9, PLINK 2.0, R, MAGMA 1.10, RVTESTS 2.1.0, GTEx v8, Coloc 5.2.3	Haplotype block estimation was performed, and single-variant X chromosome association analyses were conducted, adjusting for population structure via principal components selected by an AIC-based stepwise model. Additional analyses included APOE ε4-conditional regression, regulome-wide association, rare variant gene-based tests, and eQTL colocalization to identify shared causal variants influencing disease risk.	One risk locus (rs141773145) was identified in the female-stratified analysis in *MAP3K15*. No significant associations in the male-stratified analysis or meta-analysis.	(18)
**2025**					
Autism Spectrum Disorder	6873 cases, 8981 controls. European (100%)	GENESIS, PLINK, GWAMA, sdMAF, GATK, ANNOVAR, ERDS, CNVnator, in-house pipeline	The XWAS used PCA, logistic regression, and meta-analysis, alongside gene-sex interaction analysis. Variant calling and annotation were performed, while CNVs were detected using multiple methods; brain gene expression data were sourced from BrainSpan. A chromosome-wide significance threshold was calculated (meta-analysis p-value $< 1.51 \times 10^{-5}$; male p-value $< 7.9 \times 10^{-6}$; female p-value $< 1.07 \times 10^{-5}$).	The 59 statistically significant variants were as follows: 27 were in the male-XWAS, five in the female-XWAS, one in the combined, and nine in the meta-XWAS. Additionally, 17 variants were significant across multiple tests.	(22)

Legend: Studies as of 06/05/2025. Genome-wide significance set at p-value: 5×10^{-8} and suggestive significance set at p-value: 1×10^{-5}. Chromosome-wide significance is unique to each study and specified accordingly. AIC-based, Akaike Information Criterion; eQTL, expression quantitative trait loci; QTL, quantitative trait loci; ref, reference; SNPs, single nucleotide polymorphisms; X-APL, X-chromosome Analysis of Pedigree and Linkage. XWAS, X chromosome-wide association study

References

1. Chung RH, Ma D, Wang K, Hedges DJ, Jaworski JM, Gilbert JR, et al. An X chromosome-wide association study in autism families identifies TBL1X as a novel autism spectrum disorder candidate gene in males. Mol Autism. 2011 Nov 4;2(1):18.

2. Chu X, Shen M, Xie F, Miao XJ, Shou WH, Liu L, et al. An X chromosome-wide association analysis identifies variants in GPR174 as a risk factor for Graves' disease. J Med Genet. 2013 Jul;50(7):479–85.

3. Conde L, Foo JN, Riby J, Liu J, Darabi H, Hjalgrim H, et al. X chromosome-wide association study of follicular lymphoma. Br J Haematol. 2013 Sep;162(6):858–62.

4. Chang D, Gao F, Slavney A, Ma L, Waldman YY, Sams AJ, et al. Accounting for eXentricities: analysis of the X chromosome in GWAS reveals X-linked genes implicated in autoimmune diseases. PLoS One. 2014 Dec 5;9(12):e113684.

5. Marques CR, Costa GN, da Silva TM, Oliveira P, Cruz AA, Alcantara-Neves NM, et al. Suggestive association between variants in IL1RAPL and asthma symptoms in Latin American children. Eur J Hum Genet. 2017 Apr;25(4):439–45.

6. Lee HS, Oh H, Yang SK, Baek J, Jung S, Hong M, et al. X chromosome-wide association study identifies a susceptibility locus for inflammatory bowel disease in Koreans. J Crohns Colitis. 2017 Jul 1;11(7):820–30.

7. Zuo XY, Feng QS, Sun J, Wei PP, Chin YM, Guo YM, et al. X-chromosome association study reveals genetic susceptibility loci of nasopharyngeal carcinoma. Biol Sex Differ. 2019 Mar 25;10(1):13.

8. Hong EP, Chao MJ, Massey T, McAllister B, Lobanov S, Jones L, et al. Association analysis of chromosome X to identify genetic modifiers of Huntington's disease. J Huntingtons Dis. 2021;10(3):367–75.

9. Le Guen Y, Napolioni V, Belloy ME, Yu E, Krohn L, Ruskey JA, et al. Common X-chromosome variants are associated with Parkinson disease risk. Ann Neurol. 2021 Jul;90(1):22–34.

10. Hernangomez-Laderas A, Cilleros-Portet A, Martínez Velasco S, Marí S, Legarda M, González-García BP, et al. Sex bias in celiac disease: XWAS and monocyte eQTLs in women

identify TMEM187 as a functional candidate gene. Biol Sex Differ. 2023 Dec 11;14(1):86.

11. Romanowska J, Nustad HE, Page CM, Denault WRP, Lee Y, Magnus MC, et al. The X-factor in ART: does the use of assisted reproductive technologies influence DNA methylation on the X chromosome? Hum Genomics. 2023 Apr 21;17(1):35.

12. Hayden LP, Hobbs BD, Busch R, Cho MH, Liu M, Lopes-Ramos CM, et al. X chromosome associations with chronic obstructive pulmonary disease and related phenotypes: an X chromosome-wide association study. Respir Res. 2023 Feb 1;24(1):38.

13. Wang KW, Yuan YX, Zhu B, Zhang Y, Wei YF, Meng FS, et al. X chromosome-wide association study of quantitative biomarkers from the Alzheimer's Disease Neuroimaging Initiative study. Front Aging Neurosci. 2023 Nov 14;15:1277731.

14. Leal TP, Rao SC, French-Kwawu JN, Gouveia MH, Borda V, Bandres-Ciga S, et al. X-chromosome association study in Latin American cohorts identifies new loci in Parkinson's Disease. Mov Disord. 2023 Sep;38(9):1625–35.

15. Jantzen R, Camilleri-Broët S, Ezer N, Broët P. A statistical testing strategy accounting for random and nonrandom (skewed) X-chromosome inactivation identifies lung cancer susceptibility loci among smokers. Hum Hered. 2024 Jun 14;89(1):71–83.

16. Tangtanatakul P, Lei Y, Jaiwan K, Yang W, Boonbangyang M, Kunhapan P, et al. Association of genetic variation on X chromosome with systemic lupus erythematosus in both Thai and Chinese populations. Lupus Sci Med. 2024 Mar 8;11(1):e001061.

17. Simmonds E, Leonenko G, Yaman U, Bellou E, Myers A, Morgan K, et al. Chromosome X-wide association study in case control studies of pathologically confirmed Alzheimer's disease in a European population. Transl Psychiatry. 2024 Sep 4;14(1):358.

18. Bayram E, Reho P, Litvan I, International LBD Genomics Consortium, Ding J, Gibbs JR, et al. Genetic analysis of the X chromosome in people with Lewy body dementia nominates new risk loci. NPJ Parkinsons Dis. 2024 Feb 20;10(1):39.

19. Grunin M, Igo RP Jr, Song YE, Blanton SH, Pericak-Vance MA, Haines JL, et al. Identifying X-chromosome variants associated with age-related macular degeneration. Hum Mol Genet. 2024 Dec 6;33(24):2085–93.

20. Belloy ME, Le Guen Y, Stewart I, Williams K, Herz J, Sherva R, et al. Role of the X chromosome in Alzheimer disease genetics. JAMA Neurol. 2024 Oct 1;81(10):1032–42.

21. Le Borgne J, Gomez L, Heikkinen S, Amin N, Ahmad S, Choi SH, et al. X-chromosome-wide association study for Alzheimer's disease. Mol Psychiatry. 2025 Jun;30(6):2335–46.

22. Mendes M, Chen DZ, Engchuan W, Leal TP, Thiruvahindrapuram B, Trost B, et al. Chromosome X-wide common variant association study in autism spectrum disorder. Am J Hum Genet. 2025 Jan 2;112(1):135–53.

23. Liu Y, Li B, Deng F, Zhao X, Liu Z, Zhao J, et al. X-chromosome association study reveals genetic susceptibility loci of hypospadias in southern Chinese population. World J Urol. 2025 May 7;43(1):282.
